# DNA translocation by the CMG helicase: the helical inchworm model

**DOI:** 10.1042/BST20250145

**Published:** 2026-02-06

**Authors:** Sahil Batra, Benjamin Allwein, Y. Lucia Wang, Richard K. Hite, Dirk Remus

**Affiliations:** 1 Molecular Biology Program, Memorial Sloan Kettering Institute, New York, NY 10065, U.S.A.; 2 Structural Biology Program, Memorial Sloan Kettering Institute, New York, NY 10065, U.S.A.

**Keywords:** AAA+ ATPase, CMG, DNA helicase, DNA replication, DNA translocation, Replication fork

## Abstract

In all cells, hexameric helicases drive the unwinding of parental chromosomal DNA at replication forks to provide the single-stranded DNA templates required by replicative DNA polymerases. DNA unwinding proceeds via a steric exclusion mechanism in which the helicase encircles and translocates along one DNA strand while sterically excluding the opposite strand from its central channel. The details of how hexameric helicases translocate on single-stranded DNA remain incompletely understood and likely vary among species, as structural and mechanistic features—such as motor domain architecture and translocation polarity—shape helicase function. Recent high-resolution cryo-EM structures of the eukaryotic CMG (Cdc45–MCM–GINS) helicase, including complexes stalled at leading-strand G-quadruplexes, reveal two predominant DNA-bound conformations: planar and spiral. These structures show that different subsets of MCM subunits alternately engage the leading-strand template, defining intermediates of a nonrotary, hand-over-hand translocation mechanism. This mode of translocation differs from the sequential rotary hand-over-hand mechanism proposed for bacterial hexameric helicases, instead resembling that of other ring-shaped ATPase motors and can be described as a variant of the helical inchworm model. The evolution of this mechanism may reflect CMG’s specialized role as a replisome organizer, enabling it to coordinate accessory factors and optimize replication fork progression. Together, these findings highlight the mechanistic diversity and evolutionary adaptability of hexameric helicases.

## Introduction

Across all domains of life, rapid and efficient genome duplication prior to cell division is driven by hexameric ring-shaped DNA helicases that unwind parental duplex DNA at replication forks to provide the single-stranded DNA (ssDNA) templates necessary for replicative DNA polymerase activity. These motor proteins use ATP (or other NTP) hydrolysis to translocate directionally along DNA [1]. Despite species-specific differences, all hexameric helicases share core features. For example, ATP turnover occurs at composite active sites formed at subunit interfaces, enabling cooperative subunit activity. Moreover, the DNA substrate is threaded through the central channel via loops extending from the ATPase domains that shift axially in response to ATP binding and hydrolysis, transiently contacting the DNA to drive its passage through the channel. Ultimately, helicase translocation along ssDNA is coupled to DNA unwinding by steric exclusion of the nontracking strand from the channel.

Much of our understanding of hexameric helicase mechanisms comes from studies of helicases in bacteria, bacteriophages, and eukaryotic viruses [1,2]. In these systems, biochemical, biophysical, and structural approaches generally support a rotary hand-over-hand model in which the helicase forms a closed spiral staircase around DNA. During translocation, individual subunits advance sequentially along the helical axis, driven by sequential and coordinated ATP hydrolysis. Although the sequential rotary translocation mechanism is broadly conserved among bacterial and viral helicases, its structural details vary. For instance, in BPV E1 and SV40 large T-antigen, the DNA-binding loops spiral through out-of-plane ATPase domain rotations that maintain a planar ATPase tier [3,4], whereas bacterial DnaB and phage helicases T4 gp41 and T7 gp4 achieve the spiral via translational shifts of the ATPase domains along the channel axis [5–7]. Moreover, BPV E1 and SV40 large T-antigen coordinate 1 nucleotide of the tracking strand per subunit [3,4], whereas DnaB, T7 gp4, and T4 gp41 coordinate 2 nucleotides per subunit [5–7]. Together, these variations result in pronounced differences in the diameter and pitch of the spiral path of the ssDNA through the respective ATPase tiers.

High-resolution cryo-EM structures have recently shed light on the DNA translocation and unwinding mechanism of the eukaryotic replicative DNA helicase, CMG (Cdc45–MCM–GINS), which is composed of a hetero-hexameric ring of Mcm2-7 ATPase subunits and two noncatalytic cofactors, Cdc45 and GINS [8–14]. At first glance, these structures resemble the rotary staircase model, with the tracking strand guided in a spiral by ATPase domain loops, analogous to bacterial helicases. Structural states consistent with a rotary translocation mechanism have also been observed for archaeal homo-hexameric MCM complexes bound to ssDNA, albeit in the absence of Cdc45 and GINS orthologues [15,16]. However, unlike the strictly sequential rotary mechanism exemplified by bacteriophage T7 gp4, where mutant-poisoning experiments indicate that all subunits must be catalytically active [17–20], eukaryotic CMG tolerates active site mutations in several subunits [11,21], while the archaeal MCM helicase tolerates inactive subunits in mutant-poisoning experiments [22]. This indicates that CMG translocation on ssDNA is not obligatorily sequential. The exact underlying molecular mechanism is yet to be defined and several alternative CMG translocation models that do not invoke a strictly sequential rotary mechanism have been proposed, including asymmetric rotary [11], stochastic [23], and pumpjack [24] models.

The recent structural analysis of CMG complexes stalled at single DNA G-quadruplexes (G4s) on the tracking strand revealed previously unrecognized translocation intermediates, suggesting an alternative mechanism that bypasses sequential rotary subunit movements entirely [25]. In this model, CMG translocates along ssDNA via a nonrotary, oscillatory hand-over-hand motion of its motor domains, driven by sequential DNA binding and release around the ATPase ring. This mechanism shares features with substrate translocation mechanisms reported for ring-shaped protein unfoldases [26] and viral dsDNA packaging motors [27,28] and, by analogy to the latter, may be viewed as a variant of the helical inchworm model.

## Architecture of CMG at forked DNA

**Figure 1:**
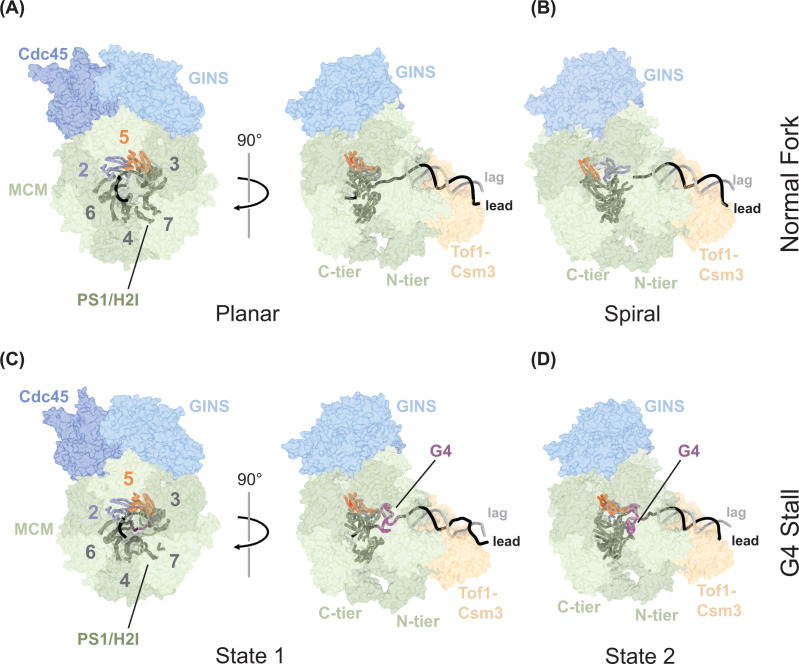
Structure of CMG bound to forked DNA Cryo-EM structure of yeast CMG in complex with Mrc1–Tof1–Csm3 bound to normal forked DNA in planar (**A**; PDB: 6SKL) and spiral (**B**; PDB: 8B9C) states or stalled at a leading strand G4 in state 1 (**C**; PDB: 9E2W) or state 2 (**D**; PDB: 9E2X). Proteins are shown as transparent surface representations; MCM PS1 and H2I loops inside the MCM central channel are highlighted.

Eukaryotic CMG consists of 11 subunits: the hetero-hexameric Mcm2-7 (MCM) complex, which serves as the catalytic core, and 2 noncatalytic components: Cdc45 and the GINS hetero-tetramer [29] (Figure 1A). The MCM subunits belong to the AAA+ family of P-loop ATPases [30]. In addition to the C-terminal ATPase domain, each MCM subunit contains an N-terminal domain (NTD) made up of three subdomains: an α-helical domain (A domain), an OB-fold, and a Zn-finger domain. Subunit-specific N- and C-terminal domains are flexibly tethered to the AAA+/NTD core [31]. The six MCM subunits assemble into a ring in the order Mcm5–Mcm3–Mcm7–Mcm4–Mcm6–Mcm2. This ring has a two-tiered architecture, with the ATPase domains forming the C-terminal tier (C-tier) and the NTDs forming the N-terminal tier (N-tier). MCM alone has weak helicase activity, which is strongly enhanced by Cdc45 and GINS [21]. This stimulation may involve stabilization of the Mcm2/5 interface that forms a gate during MCM loading [14,32–34]. In line with this role, Cdc45 and GINS associate laterally with the N-tier, bridging the Mcm2/5 and Mcm5/3 interfaces, respectively.

During DNA unwinding, CMG translocates in a 3′ to 5′ direction along ssDNA, with the N-tier oriented toward the direction of movement [12,35,36]. Duplex DNA enters the N-tier, where an invariant phenylalanine in the Mcm7 N-terminal hairpin functions as a separation pin to split the DNA, while the N-terminal hairpin loops of Mcm4 and Mcm6 guide the lagging strand template laterally upward between the Zn-finger domains of Mcm3 and Mcm5 out of the N-tier [8,13,14,37,38]. The steric exclusion of the lagging strand template from the central channel of the CMG and its specific guidance between the NTDs of Mcm3 and Mcm5 is critical to prevent premature termination via ubiquitylation-mediated CMG unloading [38–40] and to promote efficient initiation of primer synthesis on the lagging strand [37]. In contrast, the leading strand template proceeds from the N-tier into the C-tier, where it is coordinated by the presensor-1 (PS1) and helix-2-insert (H2I) hairpin loops of the MCM AAA+ domains in a spiral trajectory that slightly exceeds the diameter of B-form DNA. Each pair of PS1 and H2I loops engages ~2 nucleotides of the leading strand template, thus establishing a physical step size of ~2 nucleotides per MCM subunit.

Collectively, the reported DNA-bound CMG structures, which encompass CMG from budding yeast, fruit flies, and humans, reveal two predominant DNA-bound states: one where the leading strand template is engaged in the C-tier by Mcm3, Mcm5, Mcm2, and Mcm6 (Mcm3–5–2–6), excluding Mcm4 and Mcm7, and another where it is positioned on the opposite side of the channel to engage with Mcm4 and Mcm7 and most or all of the remaining subunits [8–14,25,37,41]. Overall, the data suggest that the leading strand template is cycled around the C-tier during translocation. In this process, CMG likely adopts two DNA-bound states that are more stable than other intermediates and, as a result, are more frequently captured in cryo-EM analyses. In contrast, the fork junction is consistently positioned in the N-tier across the reported structures, suggesting that the N-tier serves a passive structural role during DNA unwinding.

## Analysis of CMG–DNA structures reveals a nonrotary hand-over-hand mechanism

Recent studies demonstrated that leading-strand G4s impede CMG-mediated fork progression and DNA unwinding [42,43]. To uncover the structural basis of this stalling, we examined the structure of yeast and human CMG complexes stalled at a leading strand G4 by cryo-EM [25], revealing two distinct translocation states. In G4 stall state 1, the DNA is coordinated in the C-tier by Mcm3, Mcm5, Mcm2, and Mcm6, while Mcm4 and Mcm7 are unbound from DNA. In G4 stall state 2, the DNA has rotated to the opposite face of the C-tier channel, where it is coordinated by Mcm2, Mcm6, Mcm4, and Mcm7, whereas Mcm3 and Mcm5 are detached from DNA. The two G4 stall states differ by an ~180° in-plane rotation of the G4 within the N-tier: state 1 positions it toward Mcm2 and Mcm6, and state 2 toward Mcm3 and Mcm7. G4 passage into the C-tier is blocked by steric clashes with the constricting PS1 and H2I loop tips at the N-tier/C-tier interface, causing CMG stalling (Figure 1B).

**Figure 2:**
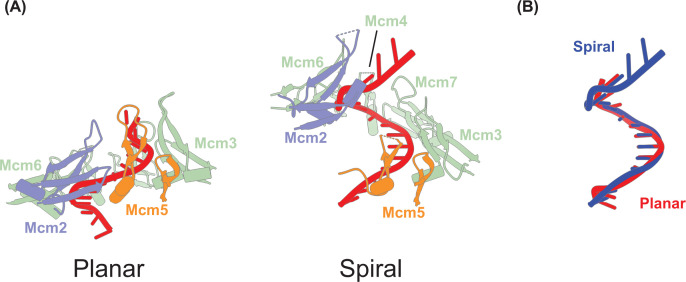
The PS1 and H2I hairpin loops predominantly adopt planar or spiral states (**A**) Planar and spiral states of the PS1 and H2I hairpin loops of yeast CMG bound to normal forked DNA (PDBs 6SKL and 6SKO) aligned on Mcm5. The leading strand template is highlighted in red. Mcm2 is highlighted in blue, Mcm5 in orange, remaining MCM subunits in green. (**B**) Superposition of the DNA coordinated in the C-tier in the spiral (blue) or planar (red) loop states.

To better understand the G4-induced CMG stall, we compared PS1 and H2I loop conformations in the G4 stall states with those in previously reported CMG-forked DNA structures [8–11,13,14,37]. Unexpectedly, this analysis revealed that the PS1 and H2I loops of CMG bound to canonical forked DNA typically adopt one of two configurations: either planar or spiral [25] (Figure 2A). The two loop conformations correspond to the major DNA-bound states described above: in the planar loop state, the DNA is coordinated by Mcm3–5–2–6, whereas in the spiral loop state, the DNA is rotated around the C-tier channel and additionally coordinated by Mcm4 and Mcm7. Notably, the path of the DNA bound to Mcm3–5–2–6 in the planar state mirrors that of the DNA bound to Mcm5–3–7–4 in the spiral state (Figure 2B). The spiral state is thus largely defined by the out-of-plane translational movement of Mcm6 and Mcm2. The recurrent capture of planar and spiral PS1/H2I loop conformations by cryo-EM suggests that these two states constitute relatively stable intermediates, likely interconnected by a continuum of transient conformational states (Supplementary Movie S1). Notably, the spiral termini are consistently defined by the PS1/H2I loops of Mcm2 and Mcm5. This recurring feature argues against a strictly sequential, rotary hand-over-hand mechanism, which would predict alternating occupancy of the spiral termini by all six MCM subunits.

Analysis of the G4 stall states offers insight into the intermediates linking the planar and spiral configurations [25]. G4 stall state 1 corresponds to the planar conformation of CMG bound to canonical forked DNA. In contrast, G4 stall state 2 adopts a conformation intermediate between the planar and spiral states, consistent with a transitional stage of the spiral-to-planar conversion. In this state, Mcm5 and Mcm3 have disengaged from DNA and transitioned 3′–5′ along the helical axis, but Mcm7 is only partially detached from DNA and Mcm4 is still fully bound to DNA. The G4 structure appears to sterically impede completion of the 3′ to 5′ movement of Mcm5 and Mcm3, thereby preventing the reestablishment the Mcm2/5 interface characteristic of the planar state. Although it is possible that G4 stall state 2 represents a stall-specific conformation not associated with normal translocation, we note that a similar state, designated state 2A, has been observed for *Drosophila* CMG imaged in the presence of ATP on canonical forked DNA, supporting the interpretation that G4 stall state 2 is a translocation intermediate [11].

## CMG translocation cycle

**Figure 3:**
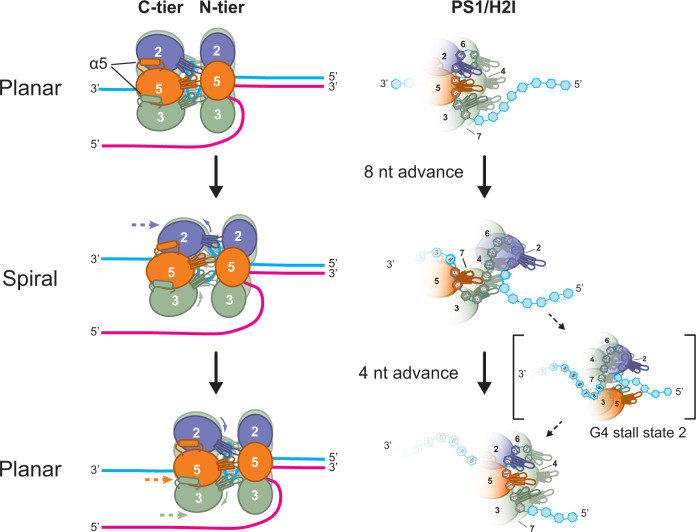
CMG transitions between planar and spiral states during translocation on ssDNA Left: Cartoon representation of CMG illustrating global conformational changes during translocation, including the movement of subunits in the C-tier and tilting of the N-tier. Mcm2 is highlighted in blue, Mcm5 in orange, remaining MCM subunits in green; cyan: leading strand template; magenta: lagging strand template; Cdc45 and GINS have been omitted for clarity. Dashed arrows indicate translational movement; small curved arrows indicate rotational movements. Right: DNA-bound states of PS1 and H2I loops in planar and spiral states illustrate the DNA translocation steps associated with the planar-to-spiral-to-planar transitions, defining one complete translocation cycle; hexagons indicate nucleotide positions; unbound substrate nucleotides are depicted in blue; bound substrate nucleotides are colored according to the respective coordinating MCM subunit; bound nucleotide numbers are in white, released nucleotide numbers (post translocation step) are in black.

The available structural data suggest that CMG translocates on ssDNA through alternating planar and spiral states as follows (Figure 3): In the planar state, Mcm6, Mcm2, Mcm5, and Mcm3 coordinate 8 nucleotides of the leading strand template (3′–5′), while Mcm7 and Mcm4 are detached from DNA. Transition to the spiral state disrupts the Mcm2/5 PS1–H2I interface, allowing Mcm2 and Mcm6 to disengage from DNA and shift toward the 5′ end, while Mcm5 and Mcm3 remain bound at the 3′ end. This shift coincides with the sequential engagement of 4 nucleotides on the 5′ side of Mcm3 by Mcm7 and Mcm4, followed by Mcm6 and Mcm2 re-engaging the next 4 nucleotides adjacent to Mcm4. Thus, the planar-to-spiral transition yields an 8-nucleotide advance by CMG. Subsequently, during the spiral-to-planar transition, Mcm5 and Mcm3 release from DNA at the 3′ end of the template and sequentially rebind 4 nucleotides at the 5′ end adjacent to Mcm2, guided by the restoration of the Mcm2/5 interface, thus adding 4 nucleotides of forward movement. During this step, Mcm7 and Mcm4 sequentially disengage from the DNA to re-establish the planar state, while Mcm6 and Mcm2 remain bound at the 5′ end of the template. Combined, the 8-nt advance during the planar-to-spiral transition and the 4-nt advance during the spiral-to-planar transition yield a 12-nt advance by CMG per translocation cycle, in line with the observed coordination of 2 nt of template DNA per MCM subunit. Notably, in this model, nonrotary motor domain movements are coupled to the sequential association and disassociation of the DNA template around the MCM AAA+ ring, raising the possibility that DNA-binding energy contributes to driving the conformational transitions in the CMG C-tier. Moreover, the sequential rotary transfer of the DNA substrate around the MCM ring likely allows CMG to maintain its grip on the DNA throughout the translocation cycle and thus enhance its processivity.

## A flexible linker domain maintains the integrity of the C-tier

**Figure 4:**
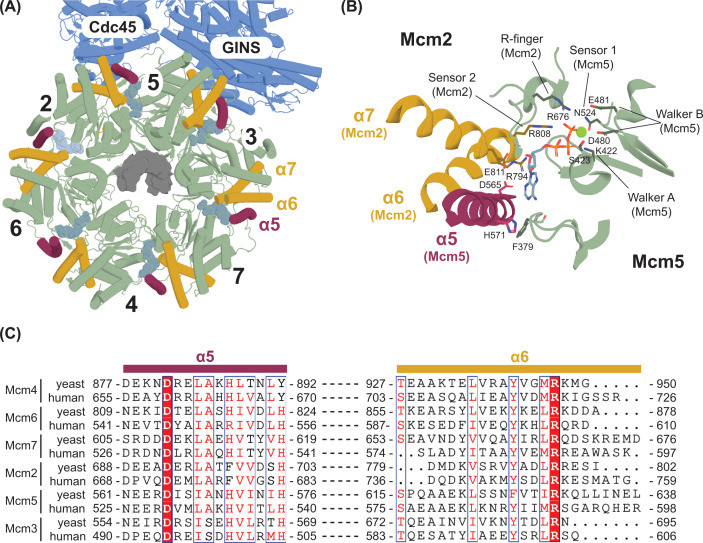
The α5 linker maintains C-tier integrity (**A**) CMG (PDB: 9E2W) viewed from 3′ end of the tracking strand highlights the domain swap by α5, which forms a helical bundle with α6 and α7 of the adjacent subunit at subunit interfaces. (**B**) The α5/6/7 bundle forms part of the *trans*-acting component of the composite ATPase sites at MCM subunit interfaces. Shown is a detailed view of the composite ATPase site at the yeast Mcm2/5 interface, with catalytic residues highlighted. (**C**) Sequence conservation of α5 and α6 across yeast and human MCMs. Conserved aspartate and arginine residues involved in salt bridge interaction between α5 and α6 are highlighted by solid red background.

PS1 and H2I loop movements (Supplementary Movie S1) are driven by coordinated rotational and translational movements of the AAA+ motor domains, coupled to oscillatory tightening and loosening of the Mcm2/5 C-tier interface (Supplementary Movie S2). These motions also periodically tilt the rigid N-tier relative to the C-tier [10,12,13]. The integrity of the C-tier during these conformational transitions is maintained by a linker helix present in all MCM subunits, corresponding to α-helix 5 (α5) of the AAA+ fold [30]. This linker helix is flexibly connected to the AAA+ α/β core and engages in a domain swap with α6 and α7 of the adjacent AAA+ domain (Figure 4A). This arrangement is facilitated by the presensor-2 α-helical insertion, which repositions α6 and α7 laterally across the AAA+ α/β core and places the catalytic sensor-2 arginine residing in α7, along with a conserved glutamate that coordinates the ATP ribose, in *trans* toward adjacent ATP-binding sites—unlike the sensor-2 motifs of members of most other AAA+ clades, which typically act in *cis* [30] (Figure 4B). Helix α6 bridges α5 and α7, in part through a conserved salt bridge formed between invariant aspartate and arginine residues in α5 and α6, respectively (Figure 4C). Thus, the α5/6/7 helix bundle, together with the adjacent R-finger domain, forms the *trans*-acting component of the composite ATP binding sites while sustaining the C-tier structure during ATP turnover.

Functionally analogous, but evolutionarily unrelated, linkers are present in spiral SF4 helicases (e.g. DnaB, G40P, T7 gp4, and T4 gp41) [5–7,44–46]. In contrast, such linkers are absent in bacterial Rho (SF5) or the eukaryotic viral helicases (SF3), BPV E1, and SV40 large T-antigen, which reposition DNA-binding loops mainly via motor domain rotation while maintaining an overall planar conformation [3,4,47]. This supports the notion that the CMG C-tier experiences global conformational rearrangements during translocation.

## ATP utilization during CMG during translocation

The mechanism by which ATP turnover drives the planar-to-spiral-to-planar oscillations of the MCM C-tier remains unresolved, as high-resolution CMG–fork DNA structures were largely captured using slowly hydrolyzable or non-hydrolyzable ATP analogs to stall the complex, which obscures normal ATP hydrolysis transition states [8–10,13,37]. On the other hand, structures obtained in ATP often lack sufficient resolution to unambiguously assign nucleotides to individual sites [11,12,14]. However, across these datasets, DNA engagement generally correlates with ATP-bound subunits, while ADP-bound or apo subunits tend to release DNA. In contrast, recent G4 stall structures captured in ATP at nucleotide-resolving resolutions reveal exceptions: ADP-bound subunits remain DNA-engaged, whereas some ATP-bound subunits disengage [25]. Structures of additional ATP-bound intermediates are therefore needed to clarify CMG’s mechanochemical coupling.

Because both spiral and planar states are observed in the presence of non-hydrolyzable ATP analogs, hydrolysis likely does not supply the mechanical force for these transitions. Instead, ATP binding may stabilize specific conformations sampled thermally by CMG, shifting their equilibrium. ATP turnover could then bias transitions directionally, functioning as a Brownian ratchet. This concept resembles the “entropy switch” model proposed for SV40 large T-antigen, where ATP binding traps high-energy conformations and hydrolysis or product release relieves this constraint to permit entropy-driven movement [4].

Importantly, mutational studies of Walker A (WA) motifs (*cis* ATP binding) and arginine-finger (RF) residues (*trans* hydrolysis) indicate that translocation involves a nonsequential ATPase cycle [11,21]. Most WA or RF substitutions cause mild or no loss of CMG helicase activity, but WA mutations in Mcm5 and Mcm3, as well as an RF mutation in Mcm3 that impairs ATP hydrolysis by Mcm7, abolish unwinding. These findings implicate Mcm5-dependent ATP turnover in regulating the Mcm2/5 interface, whose dynamics underlie planar/spiral transitions. The asymmetric clustering of essential sites near Mcm5, involving Mcm3 and Mcm7, may suggest a functional partitioning of the MCM ring into subsets of protomers that shift relative to one another during these transitions. Details of such a mechanism remain to be elucidated.

## Helical inchworm-like motor domain movements may accommodate inactive subunits

The observation that mutations in the ATP-binding sites of Mcm2, Mcm4, and Mcm6 are largely tolerated by the CMG helicase is intriguing, as all three sites are observed in both ATP- and ADP-bound states in the G4 stall states [25], indicating that they are catalytically active during translocation. How can these observations be reconciled? Insight may come from the study of orthogonal hexameric AAA+ motors, such as ClpX, a bacterial protein unfoldase involved in protein turnover [26].

Previous work demonstrated that ClpX maintains activity even in the presence of a subset of inactive protomers, which initially led to the hypothesis that ClpX acts by a stochastic instead of a sequential translocation mechanism [48]. However, subsequent studies revealed that the kinetic step size of ClpX, i.e. the movement of the substrate between successive rate-limiting steps, even with all active sites intact, significantly exceeds its physical step size. Thus, while structural data demonstrate a physical step size of 1–2 residues per ClpX protomer [49], single-molecule optical trapping studies reveal a kinetic step size of 5–7 residues [50–53]. Consistent with a larger kinetic step size, two predominant states with the substrate translocated by 6 residues between both states have been observed by cryo-EM [54]. Similarly, the Lon protease, which is related to ClpX, also translocates substrate in kinetic step sizes of 6 residues, exceeding its physical step size of 1–2 residues/subunit [55]. Moreover, Lon only hydrolyzes 1 ATP during one 6-residue step, indicating that not all subunits participate catalytically in translocation. Taken together, these observations suggest that hexameric motor subunits can undergo passive translocation in the wake of actively advancing subunits, which may explain the tolerance of hexameric motors, including CMG, to active site mutations in individual protomers. Such a mechanism would also be consistent with the concomitant advance of sets of protomers during the planar-to-spiral and spiral-to-planar transitions of CMG. Interestingly, kinetic step sizes larger than the physical step sizes have also been observed for T7 gp4, further raising the possibility that similar mechanisms operate more widely in hexameric helicases [56–58].

We note that the planar-to-spiral-to-planar transitions of the MCM C-tier in the nonrotary hand-over-hand model resemble the translocation mechanism of the distantly related, pentameric Φ29 DNA packaging motor, which transports its dsDNA substrate also by alternating between planar and spiral states in a mechanism referred to as the helical inchworm model [59,60]. In the Φ29 motor, the transitions between planar and spiral states are driven by bursts of ATP turnover across its subunits. While the bursting mechanism and pentameric structure may distinguish the Φ29 motor from CMG, the coordinated transition between planar and spiral states represents a common structural alternative to the sequential rotary model. The CMG translocation mechanism may thus be considered a variant of the helical inchworm model.

## Hexameric helicases—many solutions to one problem?

CMG’s distinct translocation mechanism likely stems from the deep evolutionary divergence of hexameric helicases, which gave rise to structural variations that may underpin mechanistic differences [1,61]. For example, bacterial helicases use RecA-type ATPase domains, whereas archaeal/eukaryotic helicases—including CMG—use AAA+ ATPase domains. Although both derive from the ASCE P-loop ATPase fold, their catalytic residues and DNA-binding loops arose from different regions of the ASCE core [1]. Moreover, the ATPase folds adopt orthogonal orientations in RecA- versus AAA+-type rings, correlating with opposite translocation polarities—5′ to 3′ for RecA-type and 3′ to 5′ for AAA+-type hexameric helicases [47,62].

Additional differences in the NTDs further influence mechanism. MCM NTDs contain α-helical, OB-fold, and Zn-finger domains that form a rigid six-fold symmetric N-tier, whereas bacterial DnaB’s α-helical hairpins dimerize to yield a three-fold symmetric, flexible N-tier that can adopt constricted, dilated and open states [7,63–65]. Viral helicases, such as BPV E1 [66] and SV40 large T-antigen [4], have yet distinct N-tier architectures, while bacteriophage T7 gp4 lacks an N-tier entirely [6].

Despite inverted translocation polarities, both bacterial and archaeal/eukaryotic hexameric helicases load with their N-tier facing the 5′ end and the C-tier facing the 3′ end of the template DNA [67]. Consequently, archaeal/eukaryotic helicases position the N-tier at the fork junction, whereas in bacteria the C-tier faces the fork, further imposing distinct mechanical constraints on DNA unwinding.

## Outlook

Collectively, the data suggest that hexameric helicases employ a range of translocation strategies tailored to their structural and functional contexts. In CMG, the reliance on a translocation mechanism centered on the dynamic regulation of the Mcm2/5 interface likely reflects architectural constraints imposed by its role as a replisome organizer, integrating helicase activity not only with DNA synthesis but also with other fork-associated processes. For example, in addition to coordinating the leading- and lagging-strand polymerases, Pol α-primase and Pol ε [8,10,37,68], which perform DNA synthesis, CMG physically interacts with proteins involved in the recycling of parental histones at replication forks, such as the histone chaperone FACT [41,69,70] and the Mrc1/claspin subunit of the FPC [71–73]. Parental nucleosome recycling also involves physical interactions between histones and the Mcm2 N-terminal tail as well as polymerases Pol α and Pol ε, which mediate histone transfer to the lagging (Mcm2, Pol α) and leading (Pol ε) strands, respectively [41,74–81]. This functional partitioning of histone transfer pathways may partly underlie the asymmetric organization of eukaryotic CMG. In addition, CMG coordinates proteins involved in a multitude of additional functions, such as cohesion establishment (e.g. Ctf4, Ctf18, and Tof1 [82–84]), checkpoint signaling (e.g. FPC and Rad53 [13,85]), and DNA topology control (e.g. Tof1 and Top1 [86,87]). These distinct interaction partners bind specific sites on CMG within replisomes, further establishing MCM subunit-specific roles and thus reinforcing the inherent structural asymmetry that differentiates eukaryotic CMG from homo-hexameric helicases. This functional specialization may explain CMG’s sensitivity to mutations in specific active sites. Whether the need for functional integration of genome maintenance pathways triggered the evolution of the eukaryotic Mcm2-7 hetero-hexamer or whether such functional asymmetry is conserved even in archaeal CMG, which features a homo-hexameric MCM ring, remains to be determined.

Notably, several replisome components appear to directly influence CMG helicase activity to promote replication fork progression, including subunits of the fork protection complex (FPC; composed of Mrc1–Tof1–Csm3 in yeast or Claspin–TIM–TIPIN in humans), as well as Mcm10 and Pol ε [10,88–90]. In addition, as observed in prokaryotic systems, helicase–polymerase coupling is thought to enhance CMG’s unwinding activity, though the molecular mechanisms underlying this stimulation may differ among systems [91–96]. Future investigations into these interactions may help elucidate CMG-specific mechanisms. Although the helical inchworm model aligns with current data, we note that detailed single-molecule kinetic measurements, together with cryo-EM snapshots of additional intermediate states, will be required to confirm this model and definitively resolve the CMG translocation mechanism.

## Perspectives

- Replicative DNA helicases are hexameric motor proteins that unwind chromosomal DNA to facilitate the copying of genomes during cell proliferation and thus play an essential role for the transmission of the genetic information across generations. However, fundamental aspects of their mechanism of DNA translocation and its coupling to ATP turnover are not understood.
- Studies on bacterial and viral helicases have suggested that hexameric helicases translocate via a rotary hand-over-hand DNA translocation mechanism in which helicase subunits move in sequential order along the helical axis of the DNA substrate. In contrast, a recent analysis of structural states of the eukaryotic replicative DNA helicase, CMG, suggests a nonrotary hand-over-hand translocation mechanism reminiscent of the helical inchworm model proposed for DNA packaging motors.
- The model proposed here makes testable predictions that may guide future studies involving single-molecule optical trapping measurements to determine the kinetic step size(s) of CMG and cryo-EM analyses of additional intermediate states to resolve the mechanochemical coupling mechanism.

## Supplementary Material

Supplementary Movie S1

Supplementary Movie S2

Supplementary File

## Open Access

Open access for this article is provided via our Subscribe to Open programme, made possible through the support of our subscribing institutions. Learn more here: https://portlandpress.com/pages/open_access_options_and_prices#conditional.

## **A** **bbreviations**

C-tier: C-terminal tier
H2I: helix-2-insert
NTD: N-terminal domain
N-tier: N-terminal tier
PS1: presensor-1
ssDNA: single-stranded DNA
dsDNA: double-stranded DNA
cryo-EM: cryogenic electron microscopy
